# Sex and age as moderators in the expression of internalizing and externalizing behaviors: insights from the Child Behavior Checklist (CBCL)

**DOI:** 10.1186/s40359-025-03529-8

**Published:** 2025-11-03

**Authors:** Roald A. Øien, Hugo Cogo-Moreira, Anders Nordahl-Hansen, Emily K. Juel, Wendy K. Silverman, Kenneth Larsen, Eli R. Lebowitz

**Affiliations:** 1 Yale School of Medicine – Child Study Center, New Haven, CT USA; 2 Department of Education, UiT – The Arctic University of Norway, Tromsø, Norway; 3 Østfold University College, Halden, Norway; 4 University of South-Eastern Norway, Kongsberg, Norway

**Keywords:** Child Behavior Checklist (CBCL), Internalizing behaviors, Externalizing behaviors, Sex differences, Age differences, Child and adolescent psychology

## Abstract

**Background:**

Children referred for anxiety disorders frequently show both internalizing and externalizing problems, but the role of sex and age in shaping these behavioral expressions remains unclear. Prior research suggests sex differences may shift across development, yet findings have been inconsistent.

**Methods:**

We analyzed data from 600 children (ages 6–17; 53.2% male) referred to a pediatric anxiety specialty clinic in the Northeastern United States between 2013 and 2021. Parents completed the Child Behavior Checklist (CBCL/6–18). Ordinary least squares regression models with an interaction term (sex × age) were estimated using Hayes’ PROCESS macro (Model 1). The Johnson–Neyman technique was applied to identify developmental periods where sex differences were significant.

**Results:**

Sex significantly moderated the association between age and CBCL scores. Boys displayed higher externalizing scores than girls between ages 6 and 9.7 years, whereas girls showed higher internalizing scores from age 10.4 years onward. Effect sizes for the interactions were modest (ΔR2 range = .03–.05).

**Conclusions:**

Findings indicate that the expression of behavioral problems among anxiety-referred youth differs by both sex and developmental stage. Early externalizing difficulties in boys and later-emerging internalizing difficulties in girls suggest that findings may inform age- and sex-sensitive approaches to assessment and intervention to age- and sex-specific trajectories in pediatric anxiety populations.

**Supplementary Information:**

The online version contains supplementary material available at 10.1186/s40359-025-03529-8.

## Introduction

### Internalizing and externalizing behaviors in youth

The study of child and adolescent psychopathology has been advanced by categorizing problem behaviors into two broad spectrums: internalizing and externalizing [1, 2]. Internalizing behaviors, characterized by inward-focused symptoms such as anxiety and depression, are often associated with social withdrawal and low self-worth [3]. Externalizing behaviors, by contrast, manifest outwardly as aggression, rule-breaking, or delinquency, and can significantly affect the child’s interpersonal functioning and school adaptation [4, 5].

### Sex differences in internalizing behaviors

Epidemiological studies consistently show that sex differences in internalizing disorders such as anxiety and depression emerge during late childhood and adolescence [6, 7]. These differences are thought to arise from a combination of biological factors (e.g., pubertal hormonal changes) and socio-cultural factors (e.g., gender roles and expectations) [8–11]. However, the precise developmental timing of these sex differences remains debated, and findings are often inconsistent.

### Sex differences in externalizing behaviors

Externalizing problems are more frequently reported in boys than girls, especially in childhood [4, 5]. Biological influences, such as testosterone levels, interact with environmental contexts, including social expectations about aggression and masculinity [12–14]. While earlier literature emphasized stable male predominance, recent research suggests that shifting cultural norms may alter these trajectories [15, 16].

### The role of the Child Behavior Checklist (CBCL)

The Child Behavior Checklist (CBCL/6–18) is a widely used parent-report measure designed to assess a child’s behavioral and emotional functioning across settings [1]. Its separate scales for internalizing and externalizing behaviors allow researchers to investigate developmental and sex-related patterns with greater specificity [17–19]. Prior work has examined CBCL subdomains, such as aggression versus rule-breaking and anxious/depressed versus withdrawn/depressed, with some evidence suggesting distinct developmental and sex-specific trajectories. However, findings remain inconsistent, and relatively little research has applied modern statistical techniques (e.g., Johnson–Neyman regions of significance) to clarify these patterns in clinically referred youth.

### Rationale for a pediatric anxiety sample

Children referred for anxiety disorders present a unique opportunity to study internalizing and externalizing behaviors. Anxiety is often comorbid with both behavioral spectrums, yet the referral context may bias how problems are expressed. For instance, some externalizing behaviors may be underrecognized in anxious youth, while internalizing symptoms may be heightened compared to community samples [20, 21]. By focusing on an anxiety-referred population, this study provides clinically relevant insights into developmental sex differences that may otherwise be obscured in general population research.

### Aims of the present study

This study aimed to examine whether sex moderates the relationship between age and internalizing/externalizing behaviors in a large pediatric anxiety sample using the CBCL. By clarifying the developmental timing of sex-specific behavioral differences, we seek to refine understanding of psychopathology trajectories and inform interventions tailored to age and sex in clinical settings.

## Methods

### Participants


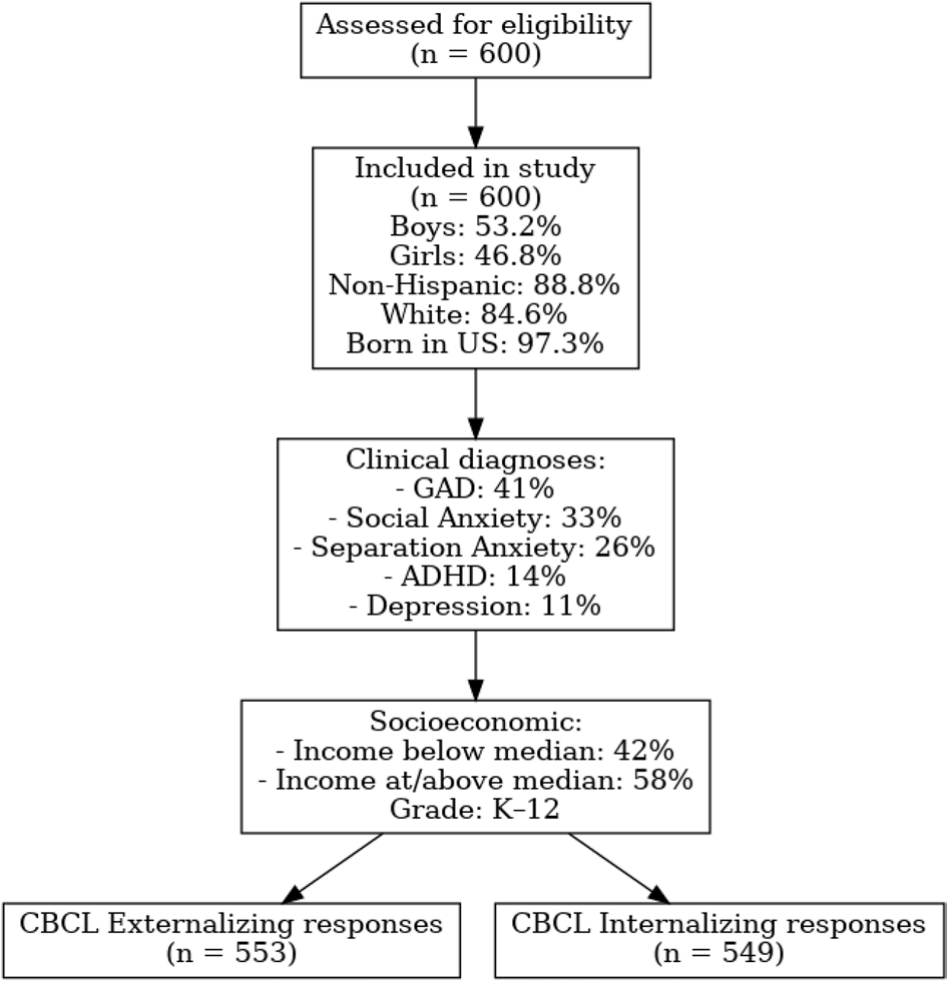


The study included 600 children aged 6–17 years (M = 11.2, SD = 3.1) referred with their mothers to a pediatric specialty anxiety disorders clinic in the Northeastern United States between 2013 and 2021. Participants were screened for eligibility through four Institutional Review Board–approved studies that shared overlapping recruitment pipelines. Inclusion criteria across the studies required: (a) primary referral for anxiety disorder evaluation; (b) age between 6 and 17 years; and (c) parent availability for assessment. Exclusion criteria included intellectual disability (IQ < 70), history of psychosis, or neurological conditions that might preclude valid assessment.

The final sample consisted of 53.2% boys and 46.8% girls. The majority were non-Hispanic (88.8%) and White (84.6%), with 97.3% born in the United States. Families represented a range of socioeconomic backgrounds, with 42% reporting household income below the state median and 58% at or above the median. The clinical sample included children with generalized anxiety disorder (41%), social anxiety disorder (33%), separation anxiety disorder (26%), and other comorbid conditions such as ADHD (14%) and depressive disorders (11%). Grade level ranged from kindergarten to 12th grade.

### Procedure

At intake, participants completed standardized diagnostic interviews (e.g., ADIS-C/P) and psychological testing to confirm anxiety disorders. Parents completed questionnaires including the CBCL/6–18. Follow-up assessments were conducted annually in the context of the four parent studies. All procedures followed the ethical standards of the Yale University IRB, and informed consent/assent was obtained from caregivers and children.

### Measures

#### Child Behavior Checklist (CBCL/6–18)

The CBCL/6–18 [1] is a standardized parent-report questionnaire designed to assess children’s behavioral and emotional functioning. It yields broadband scales for internalizing problems (withdrawn/depressed, somatic complaints, anxious/depressed) and externalizing problems (rule-breaking, aggressive behavior), as well as syndrome and competence subscales.

Internal consistency in our sample was high: Cronbach’s α = 0.89 for the problem scales and α = 0.82 for the competence scales, consistent with published reliability estimates [17].

### Statistical analysis

We tested whether the association between age and CBCL scores varied by sex using moderation models. Ordinary least squares regression models with an interaction term (age × sex) were estimated using Hayes’ PROCESS macro (Model 1) in SPSS version 26. Age was mean-centered prior to analysis, and sex was coded (0 = male, 1 = female). Both unstandardized coefficients (b) and standardized coefficients (β) are reported. Interaction effects were probed using the Johnson–Neyman technique, which identifies the range of moderator values (i.e., ages) where sex differences are statistically significant.

Missing data (< 5% across variables) were handled using listwise deletion. Covariates (race/ethnicity, SES, comorbidity) were examined but did not substantively alter the results, and final models are reported without covariates for clarity.

All analyses used a significance threshold of α = 0.05. Model assumptions were checked and met: residuals were approximately normally distributed, homoscedasticity was supported, linearity was adequate, and multicollinearity was not detected (all VIFs < 2).

## Results

The overall moderation model revealed significant effects for both domains: F_externalizing (3, 549) = 6.0081, *p* < 0.001 and F_internalizing (3, 545) = 9.1401,* p* < 0.001, meaning that the effect of age predicting each of these domains is not independent of sex. The overall interaction effect was significant for both outcomes: B_internalizing = 0.5313 (95% CI = 0.022 to 1.041) and B_externalizing = 0.5521 (95% CI = 0.077 to 1.027). In other words, given the found interaction, we cannot evaluate the main effect of age and sex independently because the effects of age on behaviors depend on specific ages.

The overall moderation models indicated significant age × sex interactions for both internalizing and externalizing behaviors. For internalizing, girls scored significantly higher than boys beginning at approximately age 10.4 and continuing through age 17.0. For externalizing, boys scored significantly higher than girls between ages 6.0 and 9.7; after this period, sex differences were no longer statistically significant. These developmental windows are summarized in Tables 1 and 2. Full Johnson–Neyman conditional effect estimates across the full age range are provided in Supplementary Tables S1–S2.

**Table 1:** Internalizing Behaviors: Johnson–Neyman Technique. *Conditional effects of sex on internalizing scores across age*

Age range (years)	Significant difference	Direction of effect
6.0–9.9	Not significant	–
10.4–17.0	Girls > Boys	Girls showed higher internalizing scores

**Table 2:** Externalizing Behaviors: Johnson–Neyman Technique. *Conditional effects of sex on externalizing scores across age*

Age range (years)	Significant difference	Direction of effect
6.0–9.7	Significant	Boys > Girls
9.9–17.0	Not significant	–

^*^Full Johnson–Neyman output (with conditional effects at each tested age) is available in Supplementary Tables S1 and S2

## Discussion

This study contributes to advancing the understanding of how sex moderates the developmental expression of internalizing and externalizing behaviors in a pediatric anxiety sample. Consistent with prior literature, our findings suggest that boys display higher levels of externalizing behaviors during early and middle childhood, particularly before the age of 10 [4, 22]. However, this pattern did not persist across later developmental stages, suggesting that the trajectory of externalizing behaviors is more complex than previously assumed. For girls, the results indicated a relative increase in internalizing behaviors after the age of 10, a pattern that is partially aligned with but also distinct from earlier research conducted in community-based or non-clinical samples [11, 23].

One of the distinctive contributions of this study is its focus on a pediatric anxiety sample, which may account for certain deviations from prior findings. Anxiety disorders are strongly linked with internalizing symptomatology, and the elevated baseline levels of anxiety in this group may delay or alter the typical sex-based patterns described in epidemiological studies [20, 24]. Our results suggest that differences between boys and girls in internalizing behaviors may emerge later in clinical populations compared with non-clinical samples, underscoring the importance of considering context when interpreting developmental trends.

The developmental shifts we observed are consistent with longitudinal findings showing that aggression and rule-breaking behaviors are more common among younger boys, whereas internalizing symptoms such as anxiety and depression become more salient in girls during adolescence [7, 22]. In our sample, externalizing behaviors decreased for boys with age, while internalizing behaviors increased for girls post-puberty. This aligns with developmental models that emphasize puberty as a critical inflection point where biological, psychological, and social factors converge to shape mental health trajectories [8–10].

The implications of these findings are significant for both clinical and educational contexts. In clinical practice, interventions may need to be tailored by age and sex. Younger boys may benefit most from early behavioral regulation strategies and parent-focused interventions, while adolescent girls may require support for coping with internalizing symptoms such as anxiety and depression. In educational settings, awareness of these distinct developmental trajectories could inform differentiated strategies for promoting mental health and social-emotional learning. As emphasized in the Stoltenberg report [15], recognizing sex-specific behavioral patterns is critical for reducing disparities and fostering inclusion.

The biological underpinnings of these developmental differences should also be considered. Hormonal changes during puberty have been well documented in relation to the emergence of internalizing symptoms, particularly in girls [8, 9]. Neurodevelopmental processes, such as the maturation of the prefrontal cortex, may contribute to the decline in externalizing behaviors in boys by enhancing impulse control and reducing risk-taking [25]. At the same time, neurotransmitter systems and stress-related physiological responses may play a role in the increased vulnerability of adolescent girls to internalizing disorders [5].

Cultural and social factors remain equally important in shaping these trends. The decline in externalizing behaviors among boys may reflect broader cultural shifts in expectations around masculinity and the expression of aggression [14]. For girls, the increase in internalizing symptoms after age 10 may be influenced by heightened academic pressures, peer expectations, and social comparisons that intensify during adolescence [26]. Gender socialization processes may further reinforce these patterns: girls are often encouraged to be reflective and relational, which can contribute to greater internalizing symptoms, whereas boys may be less socially encouraged to express vulnerability, possibly leading to reductions in overt externalizing behavior but without a parallel increase in internalizing symptoms [27, 28].

School environments may also play a critical role in reinforcing gendered patterns of behavior. The transition to middle school has been identified as a period when gender dynamics become more salient and may exacerbate stereotypical behaviors [29]. Educational systems that unintentionally reproduce gender norms may thus contribute to the divergent behavioral trajectories we observed.

Despite these contributions, several limitations should be acknowledged. First, our reliance on sex assigned at birth limits the ability to capture the influence of gender identity and expression, which are increasingly recognized as essential for understanding developmental psychopathology [30, 31]. Future studies should incorporate more nuanced measures of gender to better reflect individual experiences. Second, although our sample was relatively large and clinically well characterized, it was drawn from a pediatric specialty clinic in the Northeastern United States, which may limit generalizability to broader populations. Third, while participants were monitored longitudinally, the present analyses were cross-sectional in design, restricting inferences about trajectories over time. Finally, our reliance on parent-reported measures may introduce bias, as parental perceptions may not always align with children’s self-reports or objective assessments. Multi-informant approaches that combine parent, teacher, and child reports would provide a more comprehensive view. 

## Conclusion

In summary, sex and age jointly shaped the expression of internalizing and externalizing problems in an anxiety-referred pediatric sample. Boys showed greater externalizing problems in early childhood, while girls exhibited more internalizing problems from late childhood onward. These developmental patterns highlight the importance of considering sex-specific pathways in both research and practice. Future work should employ longitudinal, multi-informant, and gender-inclusive approaches to better map the trajectories of behavioral problems and inform tailored interventions for children and adolescents with anxiety disorders.

## Supplementary Information

Supplementary Material 1

## Data Availability

The datasets generated and analyzed during the current study are not publicly available due to institutional restrictions.
