# Supplementary material for: Sex and age as moderators in the expression of internalizing and externalizing behaviors: insights from the Child Behavior Checklist (CBCL)
Source: BMC Psychol. 2025 Nov 3;13:1211. doi: 10.1186/s40359-025-03529-8 (PMC12581308; doi:10.1186/s40359-025-03529-8)
Supplement: Supplementary file 1 — Supplementary Material 1 [file 40359_2025_3529_MOESM1_ESM.docx]

# Supplementary Material

**Supplementary Table S1**

*Johnson–Neyman Conditional Effects for Internalizing Behaviors by Age (full output)*

| Age (years) | Conditional effect (b) | Standard error | t | p value | LLCI | ULCI |
| --- | --- | --- | --- | --- | --- | --- |
| 6.0 | –0.83 | 1.32 | –0.63 | 0.530 | –3.43 | 1.77 |
| 6.6 | –0.54 | 1.21 | –0.45 | 0.656 | –2.92 | 1.84 |
| 7.1 | –0.25 | 1.10 | –0.22 | 0.823 | –2.41 | 1.92 |
| 7.7 | 0.05 | 1.00 | 0.04 | 0.964 | –1.92 | 2.01 |
| 8.2 | 0.34 | 0.91 | 0.37 | 0.713 | –1.46 | 2.13 |
| 8.8 | 0.63 | 0.84 | 0.75 | 0.455 | –1.03 | 2.28 |
| 9.3 | 0.92 | 0.79 | 1.17 | 0.244 | –0.63 | 2.47 |
| 9.9 | 1.21 | 0.76 | 1.60 | 0.111 | –0.28 | 2.71 |
| 10.4 | 1.51 | 0.76 | 1.99 | 0.047 | 0.02 | 2.99 |
| 11.0 | 1.80 | 0.78 | 2.31 | 0.022 | 0.27 | 3.33 |
| 11.5 | 2.09 | 0.83 | 2.53 | 0.012 | 0.46 | 3.72 |
| 12.1 | 2.38 | 0.90 | 2.66 | 0.008 | 0.62 | 4.14 |
| 12.6 | 2.67 | 0.98 | 2.73 | 0.007 | 0.75 | 4.60 |
| 13.2 | 2.97 | 1.08 | 2.76 | 0.006 | 0.85 | 5.08 |
| 13.7 | 3.26 | 1.18 | 2.76 | 0.006 | 0.94 | 5.58 |
| 14.3 | 3.55 | 1.30 | 2.74 | 0.006 | 1.01 | 6.10 |
| 14.8 | 3.84 | 1.41 | 2.72 | 0.007 | 1.07 | 6.62 |
| 15.4 | 4.14 | 1.54 | 2.69 | 0.007 | 1.12 | 7.15 |
| 15.9 | 4.43 | 1.66 | 2.66 | 0.008 | 1.16 | 7.69 |
| 16.5 | 4.72 | 1.79 | 2.64 | 0.009 | 1.20 | 8.24 |
| 17.0 | 5.01 | 1.92 | 2.61 | 0.009 | 1.24 | 8.79 |

*LLCI, lower limit of confidence interval; ULCI, upper limit of confidence interval.*

**Supplementary Table S2**

*Johnson–Neyman Conditional Effects for Externalizing Behaviors by Age (full output)*

| Age (years) | Conditional effect (b) | Standard error | t | p value | LLCI | ULCI |
| --- | --- | --- | --- | --- | --- | --- |
| 6.0 | –3.42 | 1.22 | –2.80 | 0.005 | –5.81 | –1.02 |
| 6.6 | –3.11 | 1.11 | –2.80 | 0.005 | –5.30 | –0.93 |
| 7.1 | –2.81 | 1.01 | –2.77 | 0.006 | –4.80 | –0.82 |
| 7.7 | –2.51 | 0.92 | –2.72 | 0.007 | –4.32 | –0.70 |
| 8.2 | –2.20 | 0.84 | –2.62 | 0.009 | –3.85 | –0.55 |
| 8.8 | –1.90 | 0.77 | –2.45 | 0.014 | –3.42 | –0.38 |
| 9.3 | –1.60 | 0.73 | –2.20 | 0.028 | –3.02 | –0.17 |
| 9.7 | –1.39 | 0.71 | –1.96 | 0.050 | –2.77 | 0.00 |
| 9.9 | –1.29 | 0.70 | –1.84 | 0.066 | –2.67 | 0.08 |
| 10.4 | –0.99 | 0.70 | –1.41 | 0.158 | –2.36 | 0.39 |
| 11.0 | –0.68 | 0.72 | –0.95 | 0.345 | –2.11 | 0.74 |
| 11.5 | –0.38 | 0.77 | –0.49 | 0.621 | –1.90 | 1.13 |
| 12.1 | –0.08 | 0.84 | –0.09 | 0.926 | –1.72 | 1.57 |
| 12.6 | 0.23 | 0.92 | 0.25 | 0.805 | –1.57 | 2.03 |
| 13.2 | 0.53 | 1.01 | 0.53 | 0.599 | –1.45 | 2.51 |
| 13.7 | 0.83 | 1.11 | 0.75 | 0.452 | –1.34 | 3.01 |
| 14.3 | 1.14 | 1.21 | 0.94 | 0.349 | –1.25 | 3.52 |
| 14.8 | 1.44 | 1.33 | 1.09 | 0.278 | –1.16 | 4.04 |
| 15.4 | 1.74 | 1.44 | 1.21 | 0.226 | –1.08 | 4.57 |
| 15.9 | 2.05 | 1.56 | 1.31 | 0.189 | –1.01 | 5.11 |
| 16.5 | 2.35 | 1.68 | 1.40 | 0.162 | –0.94 | 5.65 |
| 17.0 | 2.66 | 1.80 | 1.48 | 0.141 | –0.88 | 6.19 |

*LLCI, lower limit of confidence interval; ULCI, upper limit of confidence interval.*
